# Therapy of chronic extensor mechanism deficiency after total knee arthroplasty using a monofilament polypropylene mesh

**DOI:** 10.3389/fsurg.2022.1000208

**Published:** 2022-09-05

**Authors:** M Fuchs, C Gwinner, N Meißner, T Pfitzner, C. Perka, P. von Roth

**Affiliations:** ^1^ Orthopädische Universitätsklinik am RKU, Medizinische Universität Ulm, Ulm, Germany; ^2^ Centrum für Muskuloskeletale Chirurgie, Charité Universitätsmedizin Berlin, Berlin, Germany; ^3^ Klinik für Endoprothetik, Knie- und Hüftchirurgie, Vivantes Klinikum Spandau, Berlin, Germany; ^4^ Sporthopaedicum, Facharztzentrum für Orthopädie, Straubing, Germany

**Keywords:** extensor mechanism disruption, quadriceps tendon, patella tendon, patella fracture, synthetic mesh, marlex mesh, monofilament polypropylene mesh

## Abstract

**Introduction:**

Lesions of the quadriceps or patellar tendon after total knee arthroplasty (TKA) are a rare but serious complication which, if left untreated, can lead to loss of function of the knee joint. While acute and subacute extensor mechanism disruptions may have several causes, chronic deficiencies are often related to multiple prior revision surgeries for joint infection or aseptic TKA failure. Up to date, biological allograft reconstruction showed unsatisfying results. The use of a monofilament polypropylene mesh is a promising approach for this pathological condition. The aim of the present study was to evaluate clinical, functional and patient reported outcomes of this procedure in patients with chronic extensor mechanism deficiency.

**Materials and Methods:**

Twenty-eight patients with chronic extensor mechanism deficiency (quadriceps tendon rupture *n* = 9, patellar tendon rupture *n* = 19) after TKA were included in this retrospective study. None of the patients were lost to follow-up. Surgical reconstruction was performed at one institution between 2014 and 2020 with a monofilament polypropylene mesh (Marlex Mesh, Bard, Murray Hill, USA). The mean age at the time of surgery was 69 years. Patients presented with a mean BMI of 33 kg/m^2^. The mean follow-up period was 23 months.

**Results:**

The 2-year survivorship free of mesh revision was 89% [95% confidence interval (CI): 75% to 100%]. Three patients (11%) had to undergo revision because of mechanical mesh failure and received another polypropylene mesh. No further revisions were performed thereafter. Flexion was 87° (range, 30–120°) on average. The majority of patients (75%, 21/28) had a full active extension. The mean active extension lag after surgery was 4 degrees (range, 0–30°).

**Discussion:**

We observed a substantial improvement of extensor mechanism function. The majority of patients had full extension and showed good clinical results. A failure rate of over 50% has been published for alternative procedures. Thus, the use of the described augmentation technique represents a reasonable treatment option for chronic extensor mechanism disruptions of the patellar tendon as well as the quadriceps tendon after total knee arthroplasty. However, there might be a potentially higher risk for infection persistence in periprosthetic joint infection cases due to the presence of a foreign material.

## Introduction

Chronic disruption of the quadriceps or patellar tendon after total knee replacement is a rare but serious complication. These extensor mechanism insufficiencies are often associated with prior revision surgeries due to periprosthetic joint infection or aseptic TKA failure. If left untreated, this leads to substantial knee joint disfunction resulting in gradual patient immobilization. Multiple articles described various techniques for extensor mechanism reconstruction including direct repair, autologous semitendinosus and gracilis tendon grafts, achilles tendon and full extensor mechanism allografts (1–11). An even more invasive technique represents the use of rotational muscle flaps (12, 13). The main drawback of all the previously described techniques is the high failure rate. In 2011, the Mayo Clinic first described the use of a monofilament polypropylene mesh (Marlex Mesh, Bard, Murray Hill, USA) (14, 15). The described mesh has intentionally been utilized in general surgery (e.g. inguinal hernia repair) and gynecological surgical interventions (16–19). It offers substantial tensile strength and decreased foreign-body response compared with other synthetic materials. In contrast to other previously used mesh devices, the polypropylene mesh is completely integrated and intertwined by connective tissue (20, 21).

The initial study included the 3.5-year results of 13 patients with a chronic or subacute patella tendon disruption treated with this new technique. Promising results and good outcomes were reported for extensor mechanism injuries (15). A recent study analyzed 77 patients with subacute or chronic extensor mechanism deficiencies (27 quadriceps tendon disruptions, 40 patellar tendon disruptions, and 10 patella fractures) undergoing the same procedure (14). The survival free of mesh revision was 86% at 2-years. The vast majority (84%) of patients showed excellent functional outcomes. The authors reported a mean improvement in extensor lag of 26°. With regard to periprosthetic joint infection (PJI), Perry et al. reported good clinical outcomes for a group of 16 patients undergoing a two-stage exchange and Marlex Mesh reconstruction for infection after TKA (22). Due to the fact that extensor mechanism disruptions can be differentiated in acute, subacute, and chronic entities, the existing literature reveals a certain kind of heterogeneity, as patient cohorts mostly focus on subacute as well as chronic disruptions (14, 15). However, the vast majority of patients with PJI and extensor mechanism deficiency present with chronic disruptions (22). The aim of the present study was to examine the clinical, functional, and patient reported outcomes of patients with only chronic extensor mechanism deficiencies after TKA treated with a monofilament polypropylene mesh.

## Materials and methods

### Study design

**Figure 1:**
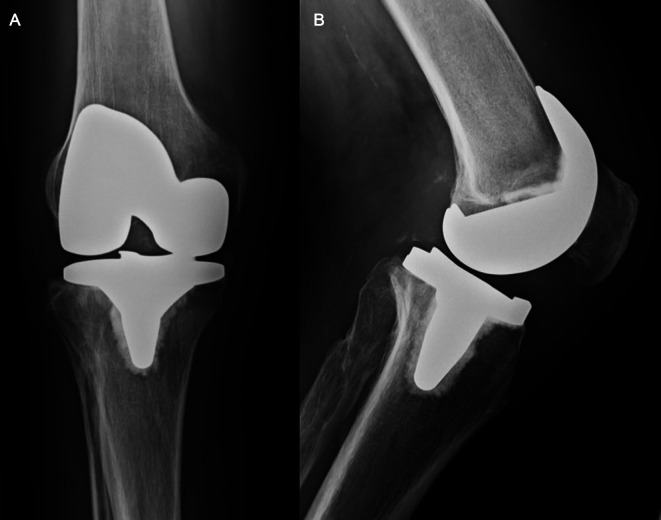
(**A,B**) Chronic quadriceps tendon insufficiency. Antero-posterior (**A**) and lateral (**B**) x-ray views of the knee of a patient with a chronic quadriceps tendon insufficiency. The patient sustained a traumatic quadriceps tendon rupture six weeks after primary TKA. Initial attempts of conservative treatment failed and lead to an extension lag of 20°, which was the indication for Marlex mesh reconstruction seven months after primary TKA.

Retrospective data collection was performed between 2014 and 2020. The study protocol was approved by the local ethics committee (registration number: EA1/035/17) and informed consent was obtained from all individuals who met the inclusion criteria which were chronic extensor mechanism disruption of either the patella- or quadriceps-tendon after previous TKA surgery. Twenty-eight patients were identified and included for further evaluation. Clinical outcomes were assessed by the Knee Injury and Osteoarthritis Outcome Score (KOOS) (23), complications and revisions were documented following an analysis of the medical records. Chronic extensor mechanism deficiency was defined as symptomatic active extension deficit of more than 10° for a time period of greater than 6 months (Figure 1). Postoperatively, full extension was defined as an extension lag not greater than 10°.

### Surgical technique and rehabilitation protocol

**Figure 2:**
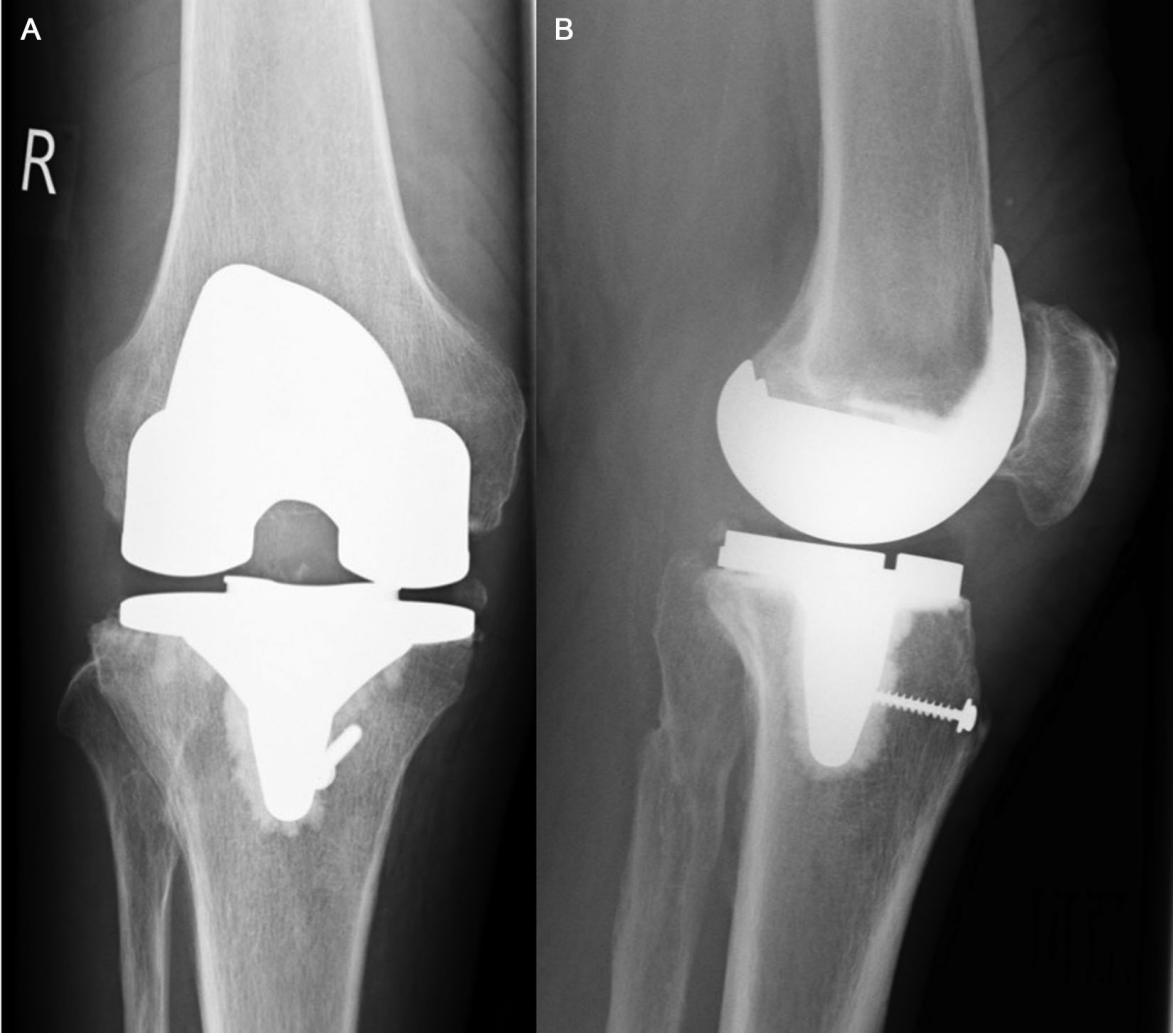
(**A,B**) Marlex Mesh fixation. Postoperative antero-posterior (**A**) and lateral (**B**) x-ray views of the knee three months after Marlex Mesh reconstruction. Regarding its distal fixation, a trough was created in the proximal-anterior aspect of the tibia. Additionally, a lag screw was placed across the mesh (as described under “2.2 Surgical Technique and Rehabilitation Protocol”).

We performed the surgical technique described earlier by Abdel and Hanssen (14, 15, 24, 25). With regard to the different options of mesh fixation, a trough was created in the proximal-anterior aspect of the tibia for the mesh to be cemented in place. Additionally, a lag screw was placed across the mesh and cemented into the host bone for additional fixation (Figures 2A,B). The mesh was then passed through a tunnel and incorporated with the remaining aspects of the host patellar tendon. At the level of the joint, it is essential to ensure that the mesh is covered with host tissue. With the limb maintained in full extension, the vastus medialis and vastus lateralis were mobilized and the mesh was sutured to the vastus lateralis, and then the vastus medialis was closed over it. Postoperatively, a stiff knee brace was applied to all patients, with the knee in full extension for 6 weeks. During this period of time, mobilization with passive flexion up to 30° was allowed. Walking was permitted with partial weight-bearing using two crutches in a knee brace locked in full extension. After 6 weeks, the knee brace was changed to an articulating brace with a range of motion from 0 to 30° but still partial weight-bearing using two crutches. Each week, range of motion (of the articulated brace) was increased by 10° until week 12. Full weight bearing was allowed at the twelfth week after surgery.

### Statistical analysis

Descriptive statistics are reported as the mean and range, or as the number and percentage. Statistical analysis was performed using SPSS, version 22 (IBM, Armonk, NY). Two-sided *P* values <0.05 were considered significant. Kaplan-Meier analysis was used to assess survivorship.

## Results

### Demographics

The study included 28 patients of which 16/28 (57%) were female. The mean age at the time of surgery was 69 years (range, 42–88 years), and the mean follow-up was 23 months (range, 5–71 months). A mean BMI of 33 kg/m^2^ (range, 23–50 kg/m^2^) was calculated. None of the patients was confirmed to be lost to follow-up. In 20 patients (71%), none of the components was revised at the time of the surgery. Those patients received an isolated reconstruction of the extensor mechanism. In 6 cases (21%), a simultaneous one-stage revision with monofilament polypropylene mesh implantation was performed. Two patients (7%) underwent extensor mechanism reconstruction during TKA reimplantation in the course of a 2-stage reimplantation procedure with previous interposition of a static spacer due to periprosthetic joint infection (PJI). Nineteen (68%) patients underwent surgery due to chronic patellar tendon disruptures, 9 (32%) individuals showed a chronic quadriceps tendon insufficiency. The surgeries were performed by three different high-volume surgeons (PR, TP, CP).

### Survivorship and complications

A total of three patients (3/28; 11%) showed a failure of the extensor mechanism reconstruction after 7, 10 and 14 months. The reason was a torn-out mesh between the vastus lateralis and the vastus medialis obliquus. All patients underwent revision surgery using the same technique. At most recent follow-up, the revised patients had no further complications and demonstrated satisfactory clinical function. The 2-year survivorship free of mesh revision was 89% [95% confidence interval (CI): 75%–100%]. No periprosthetic joint infection was observed within the study period.

### Clinical outcomes

The mean extension lag before reconstruction surgery was 35° (range, 60° to 20°). After surgery, the mean extension lag improved by 31° (range, 0°–30°) to 4° postoperatively. Seventy-five percent of patients (21/28) had full extension (between 0 and 10°) and showed good clincal results. The mean active flexion was 87° (range, 30°–120°). After surgery, patients had a mean KOOS score of 48 (range, 19–84).

## Discussion

Apart from the first descriptor’s institution, we report the largest series of patients for extensor mechanism reconstruction using a monofilament polypropylene mesh. To date, two major studies investigated the outcomes of this surgical intervention. The original article published in 2011 highlights the results of 13 patients who underwent extensor mechanism reconstruction for subacute or chronic patellar tendon disruption after TKA with an average follow-up of 42 months (15). Browne and Hanssen reported four (30.8%) early failures of graft reconstruction and nine (69.2%) patients with substantial clinical improvement at the time of final follow-up. While an extensor lag improvement of 26° (36° preoperatively compared to 10° postoperatively) was achieved, knee flexion could be maintained with an average flexion ability of 107° postoperatively. Several studies with various techniques and partly high failure rates focus on extensor mechanism reconstruction after TKA. With regard to a muscle flaps based reconstruction, Whiteside et al. reported about a residual mean extension lag of 22° (11). For allograft reconstruction techniques, a residual postoperative extension lag of 8–30° and failure rates of 15%–38% were described within the short- and medium follow-up period (4, 12, 13). Compared to these techniques, our results suggest a higher rate of successful extensor mechanism reconstruction with a mean improvement of extension lag of 31° (35° preoperatively compared to 4° postoperatively) as well as a substantial lower revision rate of 11% and an average survivorship of 89% after 2 years. These results are in accordance with the largest patient series reported by Abdel et al. (14). The authors evaluated 77 mesh reconstructions and obtained an equal survivorship free of mesh failure of 85% after 2 years. With regard to an average extensor lag improvement of 26° (35° preoperatively compared to 9° postoperatively), similar results compared to our data were documented. However, we observed a decreased active knee flexion (87°) in our study compared to the above mentioned publications [107° (15) vs. 105° (14)]. These differences might be explained by a possibly different surgical technique in terms of a higher degree of mesh tensioning in full extension prior to periarticular soft tissue integration. However, this approach may also contribute to the better extensor function of our patients postoperatively (mean extension lag 4°) compared to the reported data in the literature [mean extension lag 9° (14) and 10° (15)]. Although a marked correlation between mesh tensioning and postoperative flexion ability has so far not been investigated, this hypothesis might account for the observed clinical differences. The latter also might be a reason for the mediocre average KOOS scores (48 points) of our patients. However, these results differ from the described average KSS values of Abdel et al. of 72 points. This thesis is supported by the fact that the lowest PROM scores among our patients were documented in individuals with poor postoperative flexion abilities.

Compared to the above mentioned publications by Browne et al. and Abdel et al., our study highlights three new and important aspects regarding patient age, rehabilitation protocols and chronic extensor mechanism deficiency. First, Browne et al. and Abdel et al. dealt with a mean patient age of 60, and 65 years, respectively. In contrast, our patient population was older (69 years). With regard to the excellent results of the present study, we conclude that despite a higher patient age and the related compromised biological healing potential, synthetic mesh augmentation represents a reasonable treatment option in this group of patients. Second, we applied a different and more progressive rehabilitation protocol within the postoperative course. In our study, a stiff knee brace was applied postoperatively to all patients, with the knee in full extension for 6 weeks. During this period, mobilization with partial weight-bearing and passive flexion up to 30° was allowed. In contrast, Browne et al. described a strict knee immobilization in a long leg cast for six to eight weeks before gradual flexion exercises (15). In the study by Abdel et al. a long leg cast was applied to all patients, with the knee in full extension for even 12 weeks (14). It cannot be denied that a certain amount of immobilization is key for a successful mesh integration leading to an improvement of extensor mechanism function. However, our results reveal that also by the use of a more progressive rehabilitation protocol, excellent results in terms of extensor mechanism function can be achieved. With regard to postoperative immobilization protocols, similar outcomes were observed with either a cast immobilizer or a knee immobilizer (26). Third, this is the first study solely focusing on chronic extensor mechanism deficiencies after TKA. In this regard, the existing literature deals with chronic and subacute extensor mechanism disruptions (12, 14, 26). This raises another important issue, as the definition of chronic extensor deficiency is not consistently defined throughout the literature. Against this background, our interpretation of chronic extensor mechanism deficiency was defined as symptomatic active extension deficit of more than 10° for a time period of greater than 6 months.

PJI of the knee with concurrent disruption of the extensor mechanism is a major challenge in revision surgery. However, especially in cases of periprosthetic joint infection there is a potentially higher risk for infection persistence due to the large surface of foreign material. Perry et al. reported good clinical outcomes for a group of 16 patients undergoing a two-stage exchange and Marlex Mesh reconstruction for infection after TKA (22). At 2 years, survivorship free of PJI was 87%. The mean extensor lag improved from 31° prior to resection to 3° after mesh reconstruction. The authors concluded that two-stage exchange arthroplasty combined with Marlex Mesh reconstruction of the extensor mechanism is a viable alternative to knee arthrodesis or amputation (22). However, especially in cases of difficult-to-treat pathogens, there might be a higher risk for infection persistence due to the large surface of foreign material when using this surgical technique. This study has some limitations starting with the retrospective study design and a limited mean follow-up period of 23 months. A prolonged follow-up might lead to a decreased survivorship of the Marlex Mesh reconstruction. However, a Kaplan-Meier analysis by Abdel et al. revealed an excellent midterm survivorship free of mesh revision of 89% after 2 years which is in line with our results (89%). Second, two different extensor mechanism disruptions (patellar and quadriceps tendon) were treated with the same technique. Although this constitutes a partially heterogeneous cohort, we agree with the above-mentioned authors that this technique can be seen as an universal surgical approach for any extensor mechanism disruption. Third, a uniform definition of successful extensor mechanism reconstruction as well as a uniform definition of chronic extensor mechanism deficiency after TKA is missing throughout the existing studies. While some authors define a successful outcome as full extension or near-full extension (lag of <10°) (14), others do not consistently quantify their interpretation of treatment success with regard to a certain extensor lag threshold (12, 26, 27). Hence, a standardized and uniform classification is needed to provide a sufficient disease- and outcome-specific comparability between studies. In conclusion, the use of a monofilament polypropylene mesh for reconstructing a chronic extensor mechanism deficiency (quadriceps or patella tendon rupture) after TKA showed a good revision free survivorship. The investigated technique demonstrated substantial functional improvements and revealed a distinct decrease of complications compared to other surgical techniques. However, patients need to be informed precisely about the limited flexion ability after surgery. Additionally, in cases of PJI there might be a higher risk for infection persistence which should further be investigated.

## Data Availability

The raw data supporting the conclusions of this article will be made available by the authors, without undue reservation.
